# Martyrs to Science and the Workmen's Compensation Act

**Published:** 1911-05-06

**Authors:** 


### Martyrs to Science and the Workmen's Compensation Act.

It is not often that medical practitioners are personally concerned with claims for compensation under the Workmen's Compensation Act. The medical man appears as witness, or acts as medical referee in these cases, but it does not often occur to him that circumstances may arise in which he can himself make a claim for compensation. The report of a case in which the question arose whether a medical practitioner is, or can be, a " workman " within the meaning of the Act, is now before us. Although this point was not decided, the facts of the case were interesting in other respects.

A claim was made at the Kidderminster County Court by Cecil Frederick Curtis, the house surgeon of the Kidderminster Infirmary, for an order for compensation against the president and committee for injuries alleged to have been sustained in an accident. It appears that it was proposed to use an $x$-ray apparatus for treating certain patients suffering from ringworm. In order to test the efficacy of the apparatus, the applicant volunteered to allow it to be used on his arm, and after an exposure lasting fifteen minutes he suffered somewhat seriously. His arm had to be operated on, and he had to be away from work for six weeks. The senior honorary surgeon to the infirmary said that although the rules were silent on the point, it was obviously the duty of the house-surgeon to submit himself to the experiment. He conceded, however, that if a person submitted himself to an operation he necessarily did so of his own initiative.

In the end the Judge held that the rules imposed no duty upon the applicant to undergo this experiment, and that, therefore, the accident did not arise out of and in the course of the employment. He expressly guarded himself against deciding that the applicant was a workman within the meaning of the Act. The decisions given upon workmen's compensation are sometimes hard to understand; but this is a question of peculiar difficulty. It has been held that a workman who is injured when trying to save the life of a comrade is the victim of an accident in the course of his employment. That is what, in a modified form, Dr. Curtis would appear to have been doing. It is possible that the Court of Appeal may take a different view of the case.

